# The effect of exercise training on disease progression, fitness, quality of life, and mental health in people living with HIV on antiretroviral therapy: a systematic review

**Published:** 2015-11-30

**Authors:** Johanna Lopez, Erika Richardson, Eduard Tiozzo, Laura Lantigua, Camilo Martinez, George Abreut, Troy Prendergast, Steven E. Atlas, Andrew R. Pangilinan, Serena M. Ferris, Ana H. Martinez, Janet Konefal, Judi Woolger, Anna M. Ray, Herbert G. Simões, Carmen S.G. Campbell, John E. Lewis

**Affiliations:** 1 Department of Psychiatry & Behavioral Sciences, University of Miami Miller School of Medicine, Miami, FL, United States; 2 Department of Family Medicine, University of Miami Miller School of Medicine, Miami, FL, United States; 3 Department of Medicine, University of Miami Miller School of Medicine, Miami, FL, United States

**Keywords:** HIV/AIDS, aerobic and resistance exercise training, immune functioning, general wellbeing

## Abstract

**Background:**

Exercise has been associated with improvements in adverse physiological and psychological effects of long-term antiretroviral therapy (ART) in people living with HIV (PLWH).

**Aim:**

To summarizes the findings on the effects of aerobic or resistance training alone or combined aerobic and resistance exercise training (CARET) on disease progression, fitness, physical functioning, mental health, and quality of life (QOL) in PLWH receiving ART. A systematic search of articles was performed in several databases, and 20 articles that met inclusion criteria were summarized.

**Relevance for patients:**

Aerobic exercise was associated with improvements in aerobic capacity, QOL, and depressive symptoms, while resistance training improved strength. CARET was related to improved aerobic fitness, strength, physical functioning, QOL, and self-efficacy. At least one of the exercise interventions resulted in improvements in CD4+ cell count and HIV RNA viral load. Moreover, another study showed that HIV-specific biomarkers remained unchanged in the exercise intervention group, while they significantly worsened in the non-exercise group. In general, in spite of their well-known benefits, exercise programs have not been extensively utilized or widely recognized as viable therapeutic treatment options for this patient population. Knowing the possible health benefits of increasing physical activity level is important to better recommend exercise programs. However, the prescription must be done carefully and on an individual basis. Additional studies investigating the efficiency and effectiveness of different exercise training regimens for PLWH are needed.

## 1. Introduction

In 2011, 34 million people were living with HIV (PLWH) worldwide, of which 2.5 million represented new HIV infections [1]. The United States has about 1.1 million PLWH, and each year close to 50,000 Americans become newly infected [2, 3]. Of the 14.8 million PLWH in the world eligible for treatment in 2011, only 8 million received antiretroviral therapy (ART). Since its introduction in the mid-1990s, the administration of potent ART has resulted in a substantial reduction of HIV-associated morbidity and mortality, making HIV a chronic, but manageable illness [4-6]. With ART, most symptoms related to HIV infection such as fatigue, anorexia, wasting, cough, pain, night sweats, and fever are now manageable; however, several persistent symptoms of HIV are compounded by long-term treatment that can result in a number of unfavorable physical and psychological effects [7].

Common physical effects of ART include bloating, nausea, vomiting, diarrhea, headache, pain/neuropathy, skin rash, dry skin, anemia, lactic acidosis, osteopenia/osteoporosis, and renal, liver, and mitochondrial toxicity [4,7-9]. Furthermore, one of the most concerning adverse effects of ART is lipodystrophy, which is characterized by abnormalities in the body’s production, utilization, and distribution of fat that can result in visual atrophy of the cheeks, buttocks, and limbs, and/or may display as fat deposition on the neck and abdomen [5,10]. Lipodystrophy is associated with increased risk for diabetes mellitus and cardiovascular disease, the leading cause of death and a common cause of morbidity in Americans [5,10,11]. The risk of cardiovascular disease increases with age, regardless of HIV status, and thanks to ART, HIV patients are living longer and this, together with the side effects of ART like lipodystrophy, predisposes them to cardiovascular risks that can affect quality of life and mental health [12]. PLWH on ART have reported unfavorable psychological effects (including depression and social avoidance) as a direct result of the visibility of the lipodystrophy syndrome [13]. Other psychological responses to long-term ART regimens may include fatigue, insomnia, anxiety, agitation, confusion, nightmares, hallucinations, and mania [14]. In a survey conducted by the International Association of Physicians in AIDS Care, the majority of physicians (83.6%) believed that the most common adverse psychological responses of PLWH were a direct result of the use of ART rather than the disease itself [14, 15]. Despite breakthroughs and advances in HIV care, adverse physiological effects of ART compound persistent psychological symptoms of HIV, thus causing higher levels of distress, reduced mental health status, and lower functional quality of life (QOL) [2,7,16-18].

Today’s standard of care has shifted from treating HIV as an acute, fatal diagnosis to focusing more on the management of long-term adverse effects related to *both* HIV infection and pharmacological treatment of the disease [19]. The physical and psychological adverse reactions to ART may result in poor adherence to treatment, which typically requires daily dosing at the specified times for the remainder of the patient’s life [2]. Up to 25% of patients deviate or discontinue their therapy within the first eight months of ART initiation and consequently compromise their immunity, leading to more rapid disease progression and an inability to achieve full viral suppression [20]. Al-though medication use may ameliorate some ART-related problems, increased potential toxicities are associated with polypharmacy [21]. In addition to pharmacological treatments, effective, safe, and feasible interventions are needed to manage and prevent the anatomical/physical, metabolic, and psychological abnormalities and problems associated with HIV and ART [19].

To this point, an estimated 30-80% of PLWH in the US taking ART utilize complementary therapies to help maintain physical fitness, QOL, and positive mental health [7, 22]. For example, exercise, although not widely presented as a clinical therapeutic treatment option for PLWH, is commonly cited as one of the most accessible and highly utilized forms of selfcare among this population because of its low risk-to-benefit ratio [19,23]. More specifically, the benefits of exercise in PLWH, regardless of the type of activity, may include improvements in body composition, functional capacity, muscular strength, cognitive function, depression, anxiety, and QOL [19,24-26]. Although exercise training has been associated with positive physiological and psychological changes in PLWH, its interaction with ART represents a new area of research [7]. Given the rising prevalence of the adverse physiological and psychological consequences of long-term application of ART among PLWH, additional investigation of the therapeutic value and efficacy of exercise training is justified. Through this systematic review, we will summarize the findings of quasi-experimental studies (QES) and randomized controlled trials (RCT) on the effects of aerobic or resistance training alone or combined aerobic and resistance exercise training (CARET) on physical fitness, physical functioning, QOL, and psychosocial variables, such as depression and self-efficacy of PLWH on ART. The results of the review are intended to highlight the use of exercise and its effects on these outcome variables, leading to additional lines of research to address the multi-faceted problems of PLWH.

## 2. Methods

**Figure 1.**
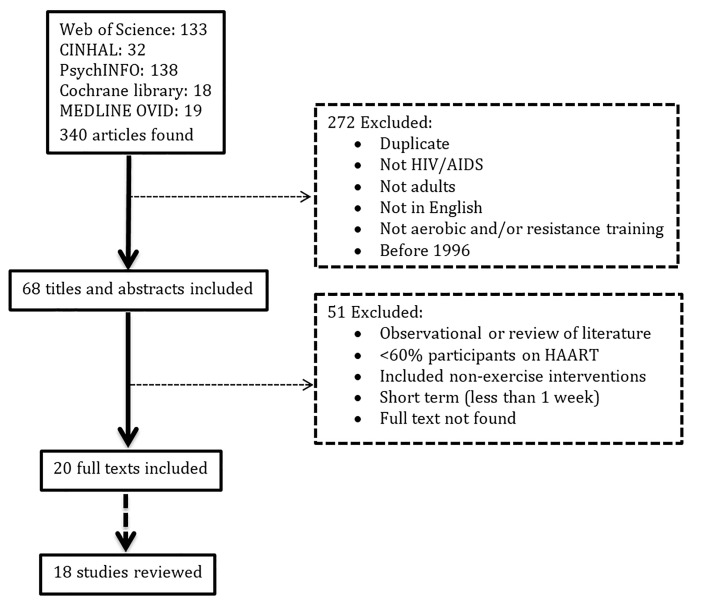
Flow chart of studies screened and excluded from systematic review

A systematic search for articles was performed using Medline Ovid, Cochrane library, PsychINFO, CINHAL, and Web of Science databases. Articles published in English between 1996 and 2015 with full texts available were searched using the terms “cardiovascular” or “aerobic” or “endurance” or “strength” or “anaerobic” or “resistance” and “exercise” or “training” and “human immunodeficiency virus” or “HIV” or “acquired immune deficiency syndrome” or “AIDS” and “antiretroviral therapy” or “ART” or “highly active antiretroviral therapy” or “HAART”. Inclusion criteria were: (a) studies describing a QES or RCT; (b) study subjects 18 years of age and older; (c) more than 60% of the subjects had to be on ART; (d) exercise intervention utilized aerobic exercise, resistance exercise, or CARET without dietary or any other therapy for ≥ 1 week; and (e) assessed outcomes related to physical and mental health pre- and post-intervention and/or compared to a non-exercise control group. Two independent reviewers evaluated the articles found for their inclusion in this review. Disagreements between the two independent reviewers were resolved by discussing them with a third reviewer. See Figure 1 for a flowchart of screening and inclusion/exclusion and Table 1 for a summary of the studies included in this review.

## 3. Results

The search resulted in 340 articles, of which 68 were identified from title and abstract prior to screening with the inclusion criteria. Full text screening of the articles identified 20 articles that met the inclusion criteria. The articles of Neidig et al. [27] and Smith et al. [28] reported different outcomes on the same study, so they were considered together as one. The same applied for the two publications by Mutimura et al. [29, 30]. Thus, 18 studies were included in this review (Table 1).

The articles found were published between 1998 and 2015. Ten RCT, two non-randomized CT, and six QES were identified that met the inclusion criteria. Although the studies by Lindegard et al. [31] and Stringer et al. [32] are randomized trials, they did not have a non-exercise control group; therefore, both exercise interventions were considered independently and only pre/post comparison within each was reported in this review. The sample size of most studies was low, ranging from 5 to 97 participants, but most of them had less than 30. The mean age of the participants in each intervention ranged from 32.2 to 53.1 years, encompassing both young adults and middle-aged adults. Twelve of the studies included men and women, while five included men only and one study included women only. Three studies evaluated the effect of resistance training, five of aerobic training, one was a crossover comparison of aerobic capacity, and nine of CARET on multiple outcomes. The most common frequency of exercise training was three times per week, while four studies used two times per week, and one study employed four times per week. The duration of the interventions ranged from six to twenty-four weeks, with most of them being sixteen weeks long. Depending on the frequency and duration, the number of sessions ranged from twelve to seventy-two, with thirty-six and forty-eight sessions being the most commonly employed. Nine studies investigated the effects of exercise on disease progression, fourteen on fitness and/or strength, two on physical functioning, three on mental health, and seven on QOL.

### 3.1. Exercise training and disease progression

Among studies that evaluated the effect of exercise on disease progression, seven of them reported no significant changes in CD4+ cell count, HIV RNA viral load, and/or both (Table 2) [27-30,32,33,33-36]. The study by Ezema and colleagues found that three weeks of moderate-intensity continuous exercise training increased VO_2_ max and CD4+ T cell count in PLWH [44]. Changes in VO_2_ max significantly correlated with changes in CD4+ T cell count (r = 0.53, p < 0.05). In addition, Tiozzo et al. [37] found a significant decrease in CD4+ T cell count (-16%, p < 0.05) in the control group, while the CARET group maintained a more stable count (-3%, p = 0.39). However, this finding was unexpected since participants were on stable ART regimens during the study; thus, adherence to medication could be a potential confounder that contributed to the difference between groups. It could be possible that the social support received by the intervention group provided a setting that promoted adherence to medications. Unfortunately, adherence to medications was not assessed in this, or any of the reviewed studies. Furthermore, a positive effect of exercise training was also shown by Stringer et al. [32], who found that those who participated in moderate aerobic training program, but not in heavy aerobic training, had an improvement in a skin test for *Candida albicans* antigen (p < 0.05), which is used to evaluate cellular immune response in those with reduced cellular hypersensitivity, when compared to the control group. Markers of disease progression remained unchanged after the interventions, so exercise training may be a safe therapeutic alternative for PLWH on ART.

### 3.2. Exercise training and aerobic capacity and strength

Aerobic fitness has been shown to be lower in PLWH (about 57-64%) in comparison to control subjects, which in turn may impair labor performance in this population [38]. Fifteen studies from the present review reported the effects of exercise training on aerobic capacity and strength (Table 2). One study revealed that VO_2_ max of PLWH increased significantly as a result of eight weeks of moderate exercise training, which was correlated with an increase in CD4+ T cell count and a decrease in blood pressure; both of potential clinical relevance [39]. Five studies evaluated the effect of aerobic training on different measures of aerobic capacity including VO_2_ max, anaerobic threshold (VO_2_ threshold), respiratory equivalent (RE), lactic acid threshold (LAT), physical endurance, and/or heart rate (Table 2). Thoni et al. [25] observed significant improvements in VO_2_ max adjusted for body weight (measured during a progressive exercise test; p = 0.005), VO_2_threshold (measured using the Beaver method; p = 0.004), and RE for oxygen (p = 0.04) following an aerobic exercise intervention. Similarly, Lindegaard et al. [31] observed a 14.4% improvement in VO_2_ max (p < 0.01) following an endurance training intervention, in addition to a 7.8% increase in strength as measured by one-repetition maximum (1-RM) calculated from 3-RM. Stringer and colleagues [32] randomized participants to a heavy or moderate progressive aerobic training intervention. They found that those in the heavy exercise group exhibited significant improvements in their peak work rate (i.e., endurance, p < 0.05), LAT (defined as the VO_2_ above which the VCO_2_ output increased faster than the VO_2_, p < 0.05) and VO _2_ max (p < 0.01), when compared to the control group. Subjects in the moderate exercise group experienced improvements in LAT only (p < 0.05) compared to the control group. Smith et al. [28] and Neidig et al. [27] found that Borg’s ratings of perceived exertion (RPE), forced expiratory volume (FEV) at one second, and VO_2_ max measurements were not significantly different between the exercise and control groups. However, endurance, as measured by time on a treadmill, improved significantly in the exercise group, when compared to the control group (p = 0.01). In the RCT by Mutimura et al. [29,30], VO _2_ max, heart rate, and RPE, as measured by the 20 meter multistage shuttle run test, significantly improved (p < 0.0001 for all) in HIV-positive participants with body fat mass redistribution who were assigned to the intervention group.

**Table 1:** Summary of all studies included in systematic review

Study #	Reference	Study design	Sample size	Sample characteristics	Intervention Description	Frequency and duration	Control
*Progressive Resistance Training*							
1	Roubenoff et al. [1]	QE	21	Mean age: 38.8 ± 7.8, 92% on ART	50% 1-RM (3 sets x 8 reps) 1^st^ session, 60% 1-RM (3 sets × 8 reps) 2^nd^ session to 75-80% 1-RM (3 sets × 8 reps) in rest of sessions.	3×/wk for 8 wks (24 sessions)	None
2	Lindegaard et al. [2] **	RCT	10	Men only with LD Mean age: 45.9 ± 8, 100% on ART	5 min. warm up; 45-60 min. 50% 1-RM (3 sets × 12 reps) to 80% 1-RM (4 sets × 8 reps)	3×/wk for 16 wks (48 sessions)	None
3	Yarasheski et al. [3]	QE	18	Asymptomatic men Mean age: 42 ± 2 100% ART	50-65% 1-RM, (2-3 sets × 10 reps) to 75-85% 1-RM (3-4 sets × 5-8 reps)	4×/wk for 16 wks (64 sessions)	None
*Progressive Aerobic Training*							
4	Stringer et al. [4]**	RCT	Total 26 CTRL8 MAT 9 HAT 9	Mean age: 36 ± 9 94% on ART	1 hr of MAT at 80% LAT, determined by CPET. Shorter time of HAT at 50% of the difference between LAT and VO_2_ MAX, equivalent to total work per session of MAT group	HAT vs. MAT 3×/wk, for 6 wks (18 sessions)	Advised to maintain current activity
5	Thoni et al. [5]	QE	Total 17	With LD Mean age: 44.2 ± 2.3, 100% ART	45 min on stationary bike at HR corresponding to VO_2_ MAX	2×/wk for 16 wks (32 sessions)	None
6	Smith et al. [6] and Neidig et al. [7]	RCT	Total 42 CTRL 24 EXS 18	Asymptomatic and symptomatic of having AIDS Mean age: 36±7 78% on ART	20 min warm up walking or jogging; 30 min of bike, stepper or cross-country machine at 60-80% of VO_2_ MAX on the GXT; 10 min cool down	3×/wk for 12 wks (36 sessions)	Waiting list
7	Lindegaard et al. [2] **	RCT	Total 8	Men with LD Mean age: 53.1 ± 8.4, 100% on ART	5 min warm up; 35 min IT at 50-100% VO_2_ MAX: 65% VO_2_ MAX first 8 wks to 75% VO_2_ MAX last 8 wks	3×/wk for 16 wks (48 sessions)	None
8	Mutimura et al. [8] and [9]	RCT	Total 97 CTRL 49 EXS 48	With BFR Mean age: 37.65 ± 2, 100% on ART	15 min warm up brisk walking; 45-60 min jogging, running, stair climbing. From 45% MHR in 1st 3 wks and 60% MHR in next 6 wks; to 75% MHR in remaining wks. Lower back and abdominal stabilization and strengthening, 15 min cool down	3×/wk for 24 wks (72 sessions)	Non-exercise, not described
9	Ezema et al. [10]	RCT	CTRL 15 EXS 15	Mean age: 38.8 (9.98) on EXS; 40.07 (9.72) on CTRL	45-60 min treadmill (60-79% HR reserve)	3×/wk, 8 wks	Conventional therapy only. Advised to maintain current activity
*CARET*							
10	Hand et al. [11]	RCT	Total 40 CTRL 19 EXS 21	Mean age: 41.8 ± 1.9 62-84% on ART	5 min warm up, 30 min AT (50-70% MHR), 20 min. RT × 12 reps, 5 min cool down	2×/wk, for 6 wks (12 sessions)	Read book, watch TV and talk at gym
11	Galantino et al. [12]	RCT	Total 38 CTRL 12 EXS 13 Tai Chi 13	Men with AIDS Age range: 20-60 100% on ART	EXS: 10 min warm up and flexibility, 15 min low impact AT at 60-70% MHR and 10 min. progressive RT Tai Chi: 10 min. seated meditation, 25 min. Chi movement of T'ai Ji drum, opening posture, looking in both directions for the healing and flying posture, 10 min healing Chi	2×/wk for 8 wks (16 sessions)	Maintain normal activities
12	Jones et al. [13]	QE	6	Mean age: 40.7 ± 13.9 100% on ART	Warm up, 20 min AT on bike at 70% MHR; and 60 min. RT, 3 sets × 10 reps upper and lower body	3×/wk for 10 wks (30 sessions)	None
13	Gomes et al. [14]	Non-randomized CT	Total 29 CTRL 10 EXS 19	Mean age: 45 ± 2 100% ART	30 min of AT on treadmill or bike at <150 bpm; 50 min of RT, 3 sets × 12 reps at 60-80% 12-RM; 10 min flexibility exercises, 2 sets × 30s at max range of motion	3×/wk for 12 wks (36 sessions)	Drop outs and waiting list
14	Tiozzo et al. [15]	RCT	Total 23 CTRL 11 EXS 12	Mean age: 45.5 ± 7 100% on ART	15-20 min AT on stationary treadmill or bicycle ergometer at 60% MHR for the first 2 wks, 5-45 min at 65% MHR the next 4 wks, 5-50 min at 70% MHR for 3 wks, and 5-50 min at 75% MHR last 3 wks. Each session: either 5-10 min of core exercises OR RT at 1-RM, 1-3 sets × 10-20 reps for 15-20 min in first 2 wks, and 20-50 min. in the remaining wks, lower and upper body exercises	3×/wk for 12 wks (36 sessions)	Telephone contact
15	Robinson et al. [16]	QE	5	With abdominal adipose tissue accumulation. Mean age: 44 ± 3.8 100% on ART	2 week pre-intervention training to reach Rx. Three progressive AT sessions per week (5 min warm up, 20 min brisk walk, jog or run on treadmill to reach 70-80% VO_2_ max, 5 min cool down) and 2 RT sessions (1 set × 8-10 reps at 60-80% 1-RM)	3×/wk for 16 s wks (48 sessions)	None
16	Dolan et al. [17]	RCT	Total 38 CTRL19 EXS 19	Women with WHR > 0.85 and BFR. Mean age: 41.5 ± 2 80-85% on ART	Supervised home based 5 min. warm up on bike at 50% MHR, flexibility exercises, 20 min AT at 60% MHR for 2 wks then 30 min. at 75% MHR for 14 wks; 20 min. RT of lower and upper body at 60% 1-RM for 2 wks 3 sets × 10, 70% 1-RM for 2 more wks 4 sets × 8, and 80% 1-RM for 10 wks 4 sets × 8	3×/wk for 16 wks (48 sessions)	Maintain normal activities
17	Roubenoff et al. [18]	QE	10	Men with self-reported increase in ab-dominal girth. Mean age: 32.2 Range: 23-56 90% on ART	20 min. AT on treadmill or stationary bicycle, 1 hr. RT at 80% 1-RM of major muscle groups of legs, back and arms. Progression based on 1-RM in proper form	3×/wk for 16 wks (48 sessions), once in supervised classes and twice on their own	None
18	Fillipas et al. [19]	RCT	Total 35 CTRL 18 EXS 17	Men only. Mean age: 43.5 ± 8.85 Range: 31-71 60-65% on ART	5 min. warm up, 20 min AT on bike, treadmill, stepper or cross trainer from 60% MHR to 75%MHR; 30 min RT on machines and with free weights for upper and lower body and core from 60% 1-RM to 80% 1-RM, 3 sets ×10 reps with 2 sec. rest period between reps and 1-2 min between sets, and 2-4 min between exercises; 5 minute cool down Progression based on RPE	2×/wk for 24 wks (48 sessions) s	20 min walking

** = Although researchers of the original study referred to it as a RCT, for the purposes of this review, it has been reported as a QE study due to its lack of control group; * = previously validated in prior HIV studies at harbor UCLA medical center; 1-RM = one repetition maximum; AIDS: acquired immunodeficiency syndrome; ART = antiretroviral therapy; AT = aerobic training; BFR = body fat redistribution; bpm = beats per minute; CPET = cardiopulmonary exercise test; CTRL = control; EXS = exercise; GXT = graded exercise test; HAT = heavy aerobic training; HR = heart rate; hr = hour; IT = interval training; LAT = lactic acid threshold defined as the VO_2_ above which the VCO_2_ output increased faster than the VO_2_; LD = lipodystrophy; MAT = moderate aerobic training; MHR = max heart rate; min = minute(s); QE = quasi-experimental; RCT = randomized controlled trial; RPE = Borg’s ratings of perceived exertion; RT = resistance training; VO_2_ MAX = maximal oxygen consumption; wks = weeks; x/wk = times per week.

**Table 2:** Summary of study results according to exercise intervention and outcome

Study #	Reference	Exercise intervention	Outcome
Disease progression			
1	Roubenoff et al. [1]	RT	NS changes in CD4 cell count or HIV RNA viral load
4	Stringer et al. [4] **	AT	NS changes in CD4 cell count and HIV RNA viral load Significant improvement of skin test for Candida albicans antigen in MAT group compared to CTRL
6	Smith et al. [6] and Neidig et al. [7]	AT	NS changes in CD4+ cell count or HIV RNA viral load
8	Mutimura et al. [8] and [9]	AT	NS changes in CD4+ cell count.
9	Ezema et al. [10]	AT	Significant increase in CD4+ cell count
13	Gomes et al. [14]	CARET	NS changes in CD4+ cell count
14	Tiozzo et al. [15]	CARET	CD4+ cell count-significant decrease in CTRL group, no change in EXS group NS changes in HIV RNA
16	Dolan et al. [17]	CARET	NS changes in CD4+ cell count and HIV RNA viral load
18	Fillipas et al. [19]	CARET	NS changes in CD4+ cell count and HIV RNA viral load
Aerobic capacity			
4	Stringer et al. [4] **	AT	Significant improvement of VO_2_ MAX, LAT, and endurance, as measured by peak work rate, in HAT group Significant improvement in VO_2_ MAX and LAT in MAT group
5	Thoni et al. [5]	AT	Significant improvement of VO_2_ MAX, VO_2_ threshold, and RE O_2_ max
6	Smith et al. [6] and Neidig et al. [7]	AT	NS improvement in RPE, FEV1 or VO2 MAX Significant improvement of endurance, as measured by time on a treadmill, compared to CTRL
7	Lindegaard et al. [2] **	AT	Significant improvement of VO_2_ MAX
8	Mutimura et al. [8] and [9]	AT	Significant improvement of VO_2_ MAX, HR, and RPE in EXS group
10	Hand et al. [11]	CARET	VO_2_ max, %FAI, peak HR, and endurance (treadmill time) significantly improved in EXS group compared to CTRL
12	Jones et al. [13]	CARET	Endurance (not specified) significantly improved
14	Tiozzo et al. [15]	CARET	VO_2_ max post intervention was significantly improved in the EXS group compared to CTRL
15	Robinson et al. [16]	CARET	NS improvement in VO_2_ max
16	Dolan et al. [17]	CARET	VO_2_ max and endurance (as measured by the submaximal bike exercise test) significantly improved in EXS compared to CTRL
18	Fillipas et al. [19]	CARET	HR (measured by Kasch pulse recovery test) significantly improved in EXS compared to CTRL
Strength			
1	Roubenoff et al. [1]	RT	Significant increase of 1-RM after intervention
2	Lindegaard et al. [2]**	RT	Significant increase of 1-RM after intervention
3	Yarasheski et al. [3]	RT	Significant increase of 1-RM after intervention
7	Lindegaard et al. [2] **	AT	Significant increase of 1-RM after intervention
12	Jones et al. [13]	CARET	1-RM significantly improved
14	Tiozzo et al. [15]	CARET	Upper and lower body 1-RM post-intervention was significantly improved in the EXS group compared to CTRL
15	Robinson et al. [16]	CARET	1-RM significantly improved for the sum of seven resistance exercises
16	Dolan et al. [17]	CARET	1-RM significantly improved in EXS compared to CTRL
17	Roubenoff et al. [18]	CARET	1-RM significantly improved
Physical functioning			
11	Galantino et al. [12]	CARET	Significant group by time interaction and test for simple main effect for time for both groups in functional reach, sit and reach, and sit up tests Significant group by time interaction in climbing one flight of stairs, climbing 3 flights of stairs
16	Dolan et al. [17]	CARET	6 min walking distance test significantly improved in EXS compared to CTRL
Mental health			
6	Smith et al. [6] and Neidig et al. [7]	AT	Decrease in depressive symptoms in EXS group as measured by CES-D and POMS No change in depressive symptoms as measured by BDI
11	Galantino et al. [12]	CARET	Significant main effect for time in confusion, bewilderment and tension anxiety for POMS Overall PMS scale was not significant SWBS improved in the 3 groups but NS compared to each other
QOL			
4	Stringer et al. [4] **	AT	Significant improvement of QOL questionnaire subset* in both groups compared to CTRL
8	Mutimura et al. [8] and [9]	AT	Significant improvement in WHOQOL-HIV BREF regarding psychological, independence, social relationships, HIV HAART specific and overall QOL in EXS group, compared to control
11	Galantino et al. [12]	CARET	MOS-HIV survey showed improvement in QOL both EXS and Tai Chi groups
13	Gomes et al. [14]	CARET	NS change in life satisfaction index
14	Tiozzo et al. [15]	CARET	SF-36 showed improvement in physical functioning and mental health post intervention in EXS compared to CTRL

** = Although researchers of the original study referred to it as a RCT, for the purposes of this review, it has been reported as a QE study due to its lack of control group;

* = previously validated in prior HIV studies at harbor UCLA medical center; 1-RM = one repetition maximum; AT= aerobic training; bpm = beats per minute; BDI = beck depression inventory; CARET = combined aerobic and resistance exercise training; CES-D = the center for epidemiological studies-depression questionnaire; CTRL = control; EXS = exercise; FEV1 **=** forced expiratory volume at 1second; GXT = graded exercise test; HAT = heavy aerobic training; HR = heart rate; hr = hour; LAT = lactic acid threshold defined as the VO_2_ above which the VCO_2_ output increased faster than the VO_2;_ MAT = moderate aerobic training; MHR = max heart rate; min = minute(s); POMS = profile of mood states depression scale; QE = quasi-experimental; QOL = quality of life; RE O_2_ max = respiratory equivalent for maximum oxygen consumption; RPE = Borg’s ratings of perceived exertion; RT = resistance training;; VCO_2_ = carbon dioxide volume;VO_2_ MAX = maximal oxygen consumption; VO_2_ threshold : oxygen consumption threshold; WHOQOL-HIV BREF = world health organization quality of life HIV short.

The three QES that evaluated the effects of progressive resistance training on strength found a significant improvement in 1-RM (Table 2) [26,31,33]. Roubenoff et al. [33] observed significant improvement in strength as measured by 1-RM in chest press, leg press, upper back, and quadriceps extension (p < 0.0001 for all) exercises for subjects who participated in the resistance training intervention. Though exercises were not specified, Lindegaard et al. [31] noted that subjects who completed a strength training intervention showed a 30% significant improvement in their strength (p < 0.0001). Yarasheski et al. [26] observed that subjects who completed a strength training intervention had a significant increase in maximum voluntary muscle strength as measured by 1-RM on all exercises including chest press, shoulder press, leg press, knee extension, and knee flexion (p < 0.001 for all).

Seven studies determined the effect of CARET on aerobic capacity and/or strength (Table 2). Four RCT and two QES reported significant changes in aerobic capacity after a CARET intervention, while one QES found non-significant changes, possibly due to the small sample size (n = 5) [35,36,40-42]. Of the four RCT that reported changes in aerobic capacity, Hand et al. [40] observed a 21% increase in VO_2_ peak (p < 0.01) and a significant improvement in endurance (i.e., treadmill time, p < 0.01), functional aerobic impairment (p value not reported), and heart rate (p < 0.05) during stages 1, 2, 4, and 6 of the graded exercise test compared to the control group. In the RCT by Tiozzo et al. [37], estimated VO_2_ max significantly improved post-intervention in the exercise group compared to the control group (+21%, p < 0.01). Dolan et al. [35] found an overall improvement in VO_2_ max (p < 0.001) and endurance (p = 0.03) as measured by the submaximal bike exercise test in the intervention group. Fillipas et al. [21] found that subjects in the CARET group had reduced heart rate (–19.6 ± 11.7 bpm), while the heart rate in the control group remained the same (0.6 ± 2.9 bpm) with a between group difference of –20.2 bpm (p < 0.001) as measured by the Kasch pulse recovery test. Only one of the QES found a significant improvement in fitness at the end of the intervention. Jones et al. [42] observed a 21.8% significant improvement in aerobic capacity (i.e., endurance, p = 0.001), while Robinson et al. [43] found non-significant changes in VO_2_ max. Despite methodological differences on aerobic fitness assessment among studies (i.e., VO_2_ max vs. VO_2_ peak or submaximal vs. maximal exercise test), all of them were consistent in measuring outcomes before and after the intervention.

The studies that evaluated the effect of CARET on strength found a significant increase in 1-RM (Table 2) [35,37,41-43]. In the Jones et al. [42] study, HIV-positive men and women experienced a 94% increases in composite strength (mean weight lifted across six different exercises, p = 0.01). Tiozzo et al. [37] observed significant improvements in upper body (+15%, p < 0.05) and lower body (+22%, p < 0.05) strength, and Dolan et al. [35] found an increase in 1-RM for knee extensors, knee flexors, ankle plantar flexors, shoulder abductors, pectorals, and elbow flexors for the exercise groups (p < 0.001 for all) in the CARET group, when compared to the control. Roubenoff et al. [33] observed an increase in 1-RM for leg press (+13%, p < 0.02), leg extension (+19%, p < 0.03), and chest press (+18%, p < 0.005), while Robinson et al. [43] found significant increases in strength as indicated by the sum of 1-RM for seven resistance exercises (lateral pull down, seated row, shoulder press, bench press, leg press, calf press, and seated leg curl; sum of all, p = 0.04).

### 3.3. Exercise training and physical functioning

Physical functioning is essential to accomplish activities of daily living and is often associated with higher perceptions of QOL [44]. Two RCT that involved CARET interventions found significant improvements in physical functioning in the intervention groups compared to the control groups (Table 2) [35, 45]. Even with only a 2 days per week intervention, Galantino et al. [45] found significant interaction and main effects for both CARET and Tai Chi groups in functional reach (p = 0.003 and p < 0.001, respectively), sit and reach (p = 0.003 and p < 0.001, respectively), and sit up tests (p = 0.001) after 8 weeks. They also found significant interactions for climbing one flight of stairs (p = 0.018) and climbing 3 flights of stairs (p = 0.018). Dolan et al. [35] found that those in the exercise group had a significant improvement in the 6-minute walk test compared to the control group (p = 0.009). We found no published studies evaluating the effect of progressive resistance training only or progressive aerobic training only on physical functioning in this population.

### 3.4. Exercise training and mental health

Depression and anxiety are the most commonly experienced symptoms in PLWH [7]. Three studies examined the effect of exercise on mental health (Table 2) [21,27,28,45]. Smith et al. [28] and Neidig et al. [27] found that progressive aerobic training significantly improved depressive symptoms as measured by Center for Epidemiological Studies-Depression Scale (p = 0.03) and Profile of Mood States (POMS; p = 0.011), but not the Beck Depression Inventory, while no changes in stress and social support were found. Galantino et al. [45] reported that CARET had a significant main effect on confusion, bewilderment, and tension anxiety on the POMS (p < 0.005), but the overall POMS scale and the Spirituality Well-Being Scale (SWBS) were not significant. Fillipas et al. [36] found that HIV-infected men who participated in 48 sessions of CARET in 24 weeks improved in self-efficacy (mean change 5.3 ± 3.7), more than the control group, with a between group difference of 6.8 (p < 0.001) (Table 2). No studies examined progressive resistance training-only interventions and their effect on mental health.

### 3.5. Exercise training and quality of life

Six studies examined the effect of aerobic training or CARET on outcomes related to QOL (Table 2) [29,30,32,34, 34-37,45]. Stringer et al. [32] observed significant changes in the aerobic training group on most questions of the subset of a QOL questionnaire, validated in prior HIV studies, compared to the control group (p < 0.01). As measured by the World Health Organization QOL HIV Short Form, Mutimura et al. [29,30] found significant improvements in psychological, independence, social relationships, HIV, and ART-specific constructs and overall QOL (p < 0.0001 for all) in the aerobic exercise arm, compared to the control group.

Four studies evaluated the change in QOL after CARET intervention (Table 2). The Medical Outcomes Study (MOS) HIV Health Survey evaluated QOL, with subscales scored from 0 to 100 and higher scores representing better health related QOL. Galantino et al. [45] observed that both exercise groups improved in the overall health perception subscale of the MOS HIV Health Survey compared to the control group (p = 0.04) after CARET intervention. Fillipas et al. [36] observed that the CARET group improved in the MOS HIV Health Survey subscales of overall health (within group difference: 14.0 ± 17.4) and cognitive functioning (within group difference: 13.5 ± 22.0), while the control group slightly reduced their overall health (within group difference: – 6.8 ± 37.5) and cognitive functioning (within group difference: – 0.5 ± 19.5) with between group differences of 20.8 (p = 0.03) and 14 (p = 0.04), respectively. Tiozzo et al. [37] utilized the SF-36 to show improvement in physical functioning (+11%, p < 0.03) and mental health (+10%, p < 0.02) at post-intervention among participants in the CARET group, when compared to the control group. Gomes et al. [34] observed pre- to post-improvements in life satisfaction, as measured by the Life Satisfaction Index, in response to CARET (p = 0.002); however, the change in the exercise group was not significantly different from the control group because life satisfaction scores were significantly different between groups at baseline. We found no articles published evaluating progressive resistance training only on QOL.

### 4. Discussion

Our review suggests that PLWH can gain significant physical and psychological benefits from aerobic and/or resistance training exercise for a minimum of 6 weeks if performed three times a week or more. The current literature supports the beneficial effects of exercise on aerobic capacity, strength, physical functioning, mental health, and QOL in PLWH. Specifically, aerobic exercise was associated with significant improvement in aerobic capacity, QOL, and depression. Resistance training was associated with improvement in strength; however, further research is needed due to the paucity of research and lack of control groups in these studies. Studies using a CARET intervention showed beneficial health outcomes in aerobic capacity, strength, physical functioning, QOL, and self-efficacy, which are positively associated with adherence to ART [36]. Overall, exercise improved fitness and/or mental health factors in most of the studies reviewed. This was expected since studies in healthy adult populations indicate that exercise not only improves cardiovascular fitness and strength but it also reduces anxiety, depression, negative mood, stress and tiredness; and improves functional capacity, autonomy, sleep, energy and stamina, self-efficacy, self-esteem, and social withdrawal [46]. The physiology and neuroscience of exercise involves increased blood flow to the brain and body as well as synthesis and release of neurotransmitters and neurotropic factors in different parts of the brain that contribute to the positive effects in mental health [47].

Based on this review, we suggest that exercise training is an adjuvant therapy that should be incorporated in the treatment plan for PLWH in order to improve fitness and mental health outcomes. Exercise seems to be a safe intervention since there was not enough evidence that indicated structured exercise training worsened or improved disease progression. Only one study revealed that eight weeks of moderate aerobic exercise training improved CD4+ T cell count of PLWH, and this increase was correlated with VO_2_ max improvement [38]. The mechanism for this outcome is unclear; however, studies in healthy populations suggest that exercise has a positive effect on several components of the innate and adaptive immune systems through multiple pathways that involve neuroendocrinological factors [48].

As for the general healthy adult population, the training prescription for PLWH should be individualized and based on prior cardiovascular and neuromuscular assessments. The immunological blood profile (i.e., CD4+ T cell count), medical treatment, and disease progression need to be considered for a better exercise prescription [12]. For adults, a suggested weekly program would include cardiovascular, strength, and flexibility training with functional exercises [12]. As in the general population, cardiovascular/aerobic training should last no more than 60min (i.e., 20-60 min of duration) at intensities of 60-75% maximal heart rate (50-60% VO_2_ max) and be performed 3-4 sessions a week, involving large muscle groups, such as walking, running, or cycling [12]. Helping practitioners choose moderate intensity exercise may be accomplished with valid scales of RPE. In addition, neuromuscular training may include both resistance and functional exercises. Free weights, stacked machines, and functional exercises should be performed 2-3 times a week with 1-2 exercises per large muscle groups and 2-3 sets initially at low intensity and high repetitions (e.g., 2 sets of 15 reps at about 50% of 1-RM each) [12]. A progressive increase in both intensity and volume of training should be possible depending on the patient’s rate of development. The inclusion of some functional exercises mimicking activities of daily living, at recreation, and/or within a patient’s work group, may prevent deterioration of autonomy and depression [12].

A similar exercise training regimen has been proposed for several special populations [12], such as individuals with type 2 diabetes, hypertension, heart disease, and the elderly. However, caution must be taken with the “dose” of exercise prescribed for the immune deficient HIV patient, especially if not on ART. Prolonged strenuous exercise of more than 1.5 hours of moderate to high intensity (55-75% of VO_2_ max) for more than one week can cause immune dysfunction characterized by bursts in respiratory neutrophils, monocyte antigen presentation, and lymphocyte proliferation in healthy and athlete populations, which could be worsened in the immune suppressed individual despite ART regimen [49]. This excessive exercise volume and intensity may result in an “open window” period in which an increased vulnerability to viruses and bacteria infection occurs. Thus, training prescription should be done carefully on an individual and regular basis, taking into account the several pathophysiological aspects of the disease and its evolution.

The studies evaluated in this review had several limitations including: (a) non-existing control groups to account for cointervention, (b) small sample sizes leading to limited interpretation of the results, and (c) inconsistency in ART treatments where not all participants within the same trial were receiving therapy and/or receiving different types of ART. Therefore, larger and longer RCT are warranted to fully elucidate the physical and psychological benefits of aerobic or resistance training or CARET in PLWH on ART.

### 5. Conclusion

Although exercise has been associated with positive physiological and psychological changes in PLWH, an exercise training program to date has not been widely utilized as a clinical therapeutic treatment option for this population. PLWH can minimize the side effects of ART by exercising regularly as part of a healthy lifestyle that also includes a proper diet, sufficient sleep, and avoiding tobacco. Since ART is the only effective treatment for HIV thus assessing interventions like exercise training programs for efficiency and effectiveness is indicated for improving the lives of PLWH. The final prescription regarding the exercise mode, its intensity, and duration must be individualized and in a multidisciplinary manner, considering the progression of the disease and its pathophysiology. In this review, some practical suggestions for exercise programing were presented, with CARET providing the most benefits in fitness and mental health outcomes and thus, warranting further research. Additional studies investigating the efficiency and effectiveness of different exercise training regimens for PLWH, mainly those on ART, are needed. In addition, more studies are needed that explore the timing of implementing exercise interventions before or during the course of ART regimen.

## List of abbreviations

1-RM: one repetition maximum
AIDS: acquired immunodeficiency syndrome
ART: antiretroviral therapy
AT: aerobic training
BDI: beck depression inventory
BFR: body fat redistribution
bpm: beats per minute
CARET: combined aerobic and resistance exercise training
CES-D: the center for epidemiological studies-depression questionnaire
CPET: cardiopulmonary exercise test
CTRL: control
EXS: exercise
FEV1: forced expiratory volume at 1 second
GXT: graded exercise test
HAART: highly active antiretroviral therapy
HAT: heavy aerobic training
HIV: human immunodeficiency virus
HR: heart rate
hr: hour
IT: interval training
LAT: lactic acid threshold defined as the VO_2_ above which the VCO_2_ output increased faster than the VO_2_
LD: lipodystrophy
MAT: moderate aerobic training
MHR: max heart rate
min: minute(s)
MOS: medical outcomes study
PLWH: people living with HIV
POMS: Profile of Mood States depression scale
QE: quasi-experimental
quasi-experimental studies: QES
QOL: quality of life
RCT: randomized controlled trials
respiratory equivalent: RE
RE O_2_ max: respiratory equivalent for maximum oxygen consumption
RPE: Borg’s ratings of perceived exertion
RT: resistance training
SF-36: short form Health survey
US: United States
VCO_2_: volume of carbon dioxide produced
VO_2_: volume of oxygen consumption
VO_2_ MAX: maximal oxygen consumption
VO_2_ peak: peak oxygen consumption
VO_2_ threshold: oxygen consumption threshold
WHOQOL-HIV BREF: World Health Organization quality of life HIV short form
wks: weeks
x/ wk: times per week
